# Editorial: Animal welfare in extensive systems

**DOI:** 10.3389/fvets.2022.1106188

**Published:** 2022-12-20

**Authors:** Severiano R. Silva, George Stilwell

**Affiliations:** ^1^Veterinary and Animal Research Centre (CECAV), Associate Laboratory of Animal and Veterinary Sciences (AL4AnimalS), University of Trás-os-Montes e Alto Douro, Vila Real, Portugal; ^2^Laboratory, Centre of Interdisciplinary Research in Animal Health (CIISA), Associate Laboratory of Animal and Veterinary Sciences (AL4AnimalS), Faculty of Veterinary Medicine, University of Lisbon, Lisbon, Portugal

**Keywords:** ruminant, extensive systems, welfare, goat, cattle

World livestock production is diverse and is supported by a wide range of systems, from intensive to extensive. Although most animal production originates from intensive systems, there are many regions in developed and developing countries where extensive systems are essential and play a role in ecosystems' integrity, social stability, cultural heritage preservation, and economic turnover. One of the most recent challenges for this system is to understand how animal welfare is impacted and how it may be assessed. To respond to this challenge, this Research Topic has put together a collection of research and review articles dedicated to a broad spectrum of topics related to animal health and welfare, as well as other features of extensive livestock production in different countries. These articles address questions about animal welfare in extensive systems, from different perspectives and for different species such as donkeys, goats, cattle, and water buffaloes.

Extensive livestock production is vital for many reasons in different continents. This aspect is discussed by Windsor in a review on the Australian reality, but with a clear message for other countries. This review emphasizes the need for continuing improvements in animal welfare to ensure social acceptance of animal-sourced food and fiber; a pertinent issue in the social context of strong opposition toward animal production. In addition, the need for sustainability of animal production is also highlighted in how it impacts the Australian economy. Severe drought periods subsequent to global climate changes have dramatically affected animal welfare in these systems, triggering the emergence of unforeseen disease severity (e.g., paratuberculosis) and overwhelming bushfires, causing an increase in mortality, morbidity, and suffering of extensive-farmed animals. This review suggests innovations for animal welfare surveillance and assessment that will improve the management of extensive farm animal welfare in Australia and will serve as a lesson globally.

Likewise, practices that will allow for the adaptation, or that will help mitigate the effects of climate change, ensuring a more sustainable production in Enugu State, Nigeria, are analyzed by Nwobodo et al. This work studies factors that significantly influenced the use of sustainable practices by 96 ruminant farmers. Access to veterinary services, monthly household, and annual income from ruminant production were considered the most important factors influencing the implementation of these practices.

Publications related to the health and welfare of donkeys are very much needed. The article by Deng et al. highlights farm demographics as well as the health and welfare issues of donkeys in Northeastern China. In this work, it is pointed out that 40% of the donkeys suffered from at least one health problem; the most common problems were colic, respiratory disorders, and skin conditions. The article also indicates that owners underestimated some of the most prevalent diseases in donkeys, which suggests that there is still room for improvement in health management, ensuring better welfare of donkeys in those regions.

The work presented by Nenadović et al. studies the impact of parasitological infections on the welfare of native goat breeds in extensive Serbian farming systems. Using the Animal Welfare Indicators (AWIN) protocol, correlations were found between infection by certain parasites and animal welfare-based indicators, such as poor hair coat condition and nasal discharge. Also working with goats, Battini et al. test the feasibility and reliability of the AWIN welfare assessment protocol for dairy goats in semi-extensive farming conditions. Inter-observer reliability analysis of different indicators showed a wide range of values, from excellent to insufficient. The results identified as insufficient were associated with differences in the background of the assessors and feasibility constraints, which is why there is a need for comprehensive training and validation of some group-level animal-based indicators, particularly those that evaluate daily activities' synchrony.

Aspects related to the behavior and welfare of cattle are addressed in three articles. Nakajima et al. show the importance of temperament trait changes in Japanese Black cows under grazing and confined conditions. The results show that grazing enhanced the cows' docility while being managed. On the other hand, Vicic et al. analyze the barriers facing non-replacement male calves in the Australian dairy industry. The main barriers identified were related to the cost and availability of feed, the additional cost of labor, and a lower economic return on the meat produced by this type of animal. Identifying these barriers represents a step toward non-replacement male calves being seen as a profitable commercial practice. Slayi et al. analyze grass species and their distribution patterns, and their effect on the behavior and weight gain of Nguni (NG) and Boran (BR) cattle, post-relocation to a novel environment. It was found that both breeds showed a reduction in weight gain and body condition in the first 3 weeks after moving, which was followed by adaptation to the novel environment conditions and stress reduction, with the recovery of their behavioral activities and weight gains.

Lastly, the article presented by Vilela et al. is related to thermolysis and skin microstructure dynamics in water buffaloes reared in a humid tropical climate. This work expands the knowledge on the heat tolerance capacity of Murrah buffaloes in tropical environments. It was shown that, despite the tolerance capacity of this species to heat stress, access to shade in buffalo rearing systems in tropical regions is essential.

In summary, the Research Topic of these articles will contribute to increasing the knowledge regarding the welfare of animals kept in more natural settings.

## Author contributions

Both authors listed have made a substantial, direct, and intellectual contribution to the work and approved it for publication.

